# The prevention, detection and management of cancer treatment-induced cardiotoxicity: a meta-review

**DOI:** 10.1186/s12885-015-1407-6

**Published:** 2015-05-07

**Authors:** Aaron Conway, Alexandra L McCarthy, Petra Lawrence, Robyn A Clark

**Affiliations:** 1 School of Nursing, Institute of Health and Biomedical Innovation, Queensland University Technology, Kelvin Grove Campus, Kelvin Grove, QLD 4059 Australia; 2 Division of Cancer Services, Princess Alexandra Hospital and School of Nursing, Institute of Health and Biomedical Innovation, Queensland University Technology, Kelvin Grove Campus, Kelvin Grove, QLD 4059 Australia; 3 Nursing Research & Practice Development Unit The Prince Charles Hospital and School of Nursing, Midwifery and Paramedicine, Australian Catholic University, Brisbane, QLD Australia; 4 School of Nursing and Midwifery, Flinders University, 5042 GPO Box 2100,, Sturt Road, Bedford Park, Adelaide, 5001 South Australia

**Keywords:** Heart failure, Chemotherapy, Cardiotoxicity, Cancer, Systematic review, Meta-review

## Abstract

**Background:**

The benefits associated with some cancer treatments do not come without risk. A serious side effect of some common cancer treatments is cardiotoxicity. Increased recognition of the public health implications of cancer treatment-induced cardiotoxicity has resulted in a proliferation of systematic reviews in this field to guide practice. Quality appraisal of these reviews is likely to limit the influence of biased conclusions from systematic reviews that have used poor methodology related to clinical decision-making. The aim of this meta-review is to appraise and synthesise evidence from only high quality systematic reviews focused on the prevention, detection or management of cancer treatment-induced cardiotoxicity.

**Methods:**

Using Cochrane methodology, we searched databases, citations and hand-searched bibliographies. Two reviewers independently appraised reviews and extracted findings. A total of 18 high quality systematic reviews were subsequently analysed, 67 % (n = 12) of these comprised meta-analyses.

**Results:**

One systematic review concluded that there is insufficient evidence regarding the utility of cardiac biomarkers for the detection of cardiotoxicity. The following strategies might reduce the risk of cardiotoxicity: 1) The concomitant administration of dexrazoxane with anthracylines; 2) The avoidance of anthracyclines where possible; 3) The continuous administration of anthracyclines (>6 h) rather than bolus dosing; and 4) The administration of anthracycline derivatives such as epirubicin or liposomal-encapsulated doxorubicin instead of doxorubicin. In terms of management, one review focused on medical interventions for treating anthracycline-induced cardiotoxicity during or after treatment of childhood cancer. Neither intervention (enalapril and phosphocreatine) was associated with statistically significant improvement in ejection fraction or mortality.

**Conclusion:**

This review highlights the lack of high level evidence to guide clinical decision-making with respect to the detection and management of cancer treatment-associated cardiotoxicity. There is more evidence with respect to the prevention of this adverse effect of cancer treatment. This evidence, however, only applies to anthracycline-based chemotherapy in a predominantly adult population. There is no high-level evidence to guide clinical decision-making regarding the prevention, detection or management of radiation-induced cardiotoxicity.

**Electronic supplementary material:**

The online version of this article (doi:10.1186/s12885-015-1407-6) contains supplementary material, which is available to authorized users.

## Background

Numerous factors, such as the introduction of screening programs to facilitate early detection [1, 2], improved diagnostic imaging, advances in therapy and the implementation of multidisciplinary cancer care [3], have contributed to improved cancer survival rates over recent decades [4, 5]. Advances in chemo- and radiotherapy have had the most impact on cancer survival [6]. The benefits associated with some cancer treatments, however, do not come without risk. A devastating side effect of some common cancer treatments is cardiotoxicity-principally heart failure. The risk of cardiotoxicity varies according to the type and intensity of cancer treatment. Heart failure incidence rates associated with the commonly-prescribed chemotherapy agents include 0.14–48 % for anthracyclines (estimated risk for doxorubicin dose > 400 mg/m [2] ranges from 0.14 % to 5 %; for 550 mg/m2 it ranges from 7 % to 26 %, and for 700 mg/m^2^ the estimated risk ranges from 18 % to 48 %) [7]. For high dose cyclophosphamides the risk ranges from 7 to 28 % for high-dose cyclophosphamides [8]. The risk is 1 % for trastuzamab (while 5 % of patients develop systolic dysfunction, only 1 % develop symptomatic cardiomyopathy) [7, 9]; and 8 to 12.5 % for tyrosine kinase inhibitors [10, 11]. Cardiotoxicity, which can occur up to 20 years after treatment [12, 13] is likely to become even more prevalent as the cancer population ages and novel, so-called ‘targeted’ treatment regimens that cause damage to cardiac myocytes are more commonly employed. Concomitant chest irradiation in blood, breast and lung cancers is also implicated in cardiotoxicity [14].

Growing recognition of the longer-term public health implications of this problem, which is expected to increase as more people successfully complete acute cancer treatment, has resulted in a great deal of research in this field. Two key strategies are commonly utilised to support implementation of evidence into clinical practice; clinical practice guidelines and literature reviews (including both systematic and non-systematic review methodology). Guidelines for preventing, monitoring and treating cancer treatment-induced cardiotoxicity are available [8]. Non-systematic reviews have been published to support clinical practice and research related to cancer treatment-induced cardiotoxicity [15]. In addition, a number of systematic reviews have been published on this issue. However, critical appraisal and synthesis of systematic reviews and meta-analyses is needed in order to ensure that decision-making is informed by the best available accumulated evidence [16]. The ‘meta-review’ employs a unique review methodology in which the findings presented in individual systematic reviews and meta-analyses are appraised and synthesized. Methods similar to a traditional systematic review, such as comprehensive literature searches and quality assessment by two reviewers, are used. The difference between a traditional systematic review, which may or may not also incorporate meta-analysis, is that a meta-review only considers results reported in systematic reviews and meta-analyses, not results from individual studies. We conducted a meta-review of the systematic reviews and meta-analyses that have addressed the important issue of cancer treatment-induced cardiotoxicity. Our aim was to appraise and synthesise the systematic reviews that have focused on the prevention, early detection and management of cancer treatment-induced cardiotoxicity in order to aid policy and practice decision-making.

## Methods

Cochrane methodology was used to appraise and synthesise systematic reviews in this field [6]. Our meta-review included a comprehensive literature search. The relevant reviews identified were then analysed by categorising and comparing the populations, interventions, comparisons and outcomes that were reported for each review. In addition, the quality of each review was appraised using a validated tool [16].

### Information sources and search strategy

The following databases were searched: CINAHL; Cochrane Database of Systematic Reviews; Joanna Briggs Institute library of systematic reviews; EMBASE; Health source nursing/academic edition; and MEDLINE. The database searches were supplemented with manual searching of reference lists plus a forward citation search using Google Scholar. Only reviews published in peer-reviewed journals were included in this review [17]. Census dates from January 1996 and October 2013 (inclusive) were set for all literature searches. Only articles written in full-text English were included [18]. Potentially relevant publications were retrieved in full-text for review purposes. The search used Boolean operators to combine free text terms and/or MeSH terms including cardiotoxicity and systematic review. An example of the search terms used in one of the databases searched is presented in Additional File 1.

### Study selection

Titles and abstracts were screened to eliminate irrelevant articles. Potentially eligible publications were retrieved and the full text version was reviewed in detail. Two reviewers independently selected studies for inclusion with a third independent reviewer was available for arbitration. Inclusion and exclusion criteria for this meta-review are outlined in Table 1.

**Table 1:** Inclusion and exclusion criteria for systematic reviews in this meta-review

**Inclusion criteria**	**• Study type:** Systematic review of original research (as per the PRISMA statement. A systematic review was defined as a review with a clearly formulated question that used systematic and explicit methods to identify, select and critically appraise relevant research and to collect and analyse data from the studies that were included in the review. As such, the review had to describe a detailed search of the literature for relevant studies and synthesis of results)
	**• Publication:** Full peer-reviewed publication
	**• Population:** Patients with cancer
	**• Intervention:** Any intervention applied to prevent, diagnose or manage cancer treatment-induced cardiotoxicity.
	**• Comparison:** Any comparison.
	**• Outcome:** Cardiotoxicity, as defined by the authors of the original systematic review. Could be clinical diagnosis of heart failure, heart failure graded by a standardized reporting system, subclinical heart failure (identified by myocardial biopsy, non-invasive imaging techniques or biomarkers) or adverse cardiac events (myocardial infarction, arrhythmia).
**Exclusion criteria**	• Systematic reviews focused on identifying the incidence of cardiotoxicity associated with particular cancer treatment regimens.
	• Poor quality (Literature search was not comprehensive, quality of included studies was not appraised, total AMSTAR score <7)

### Data extraction

In addition to extracting data to describe the characteristics of each systematic review, such as the number of studies included, year of publication and the total number of participants, data about the populations, interventions, comparisons and outcomes were extracted. These data were extracted with a standardised form developed specifically for this study by two reviewers.

### Quality appraisal

All potentially relevant reviews were appraised by two independent reviewers for their quality and risk of bias using the validated AMSTAR tool [16]. The 11 items of the AMSTAR were developed by building on empirical data collected with previously developed tools and with expert opinion. As such, the AMSTAR provided a valid, standardised method to assess the quality of methods used to search the literature and combine results, as well as the comprehensiveness that results of the reviews were reported [7]. Importantly, the AMSTAR criteria also provided a standardised method to determine the extent to which the scientific quality of the studies was assessed in the systematic reviews. The Cochrane Collaboration specify this as an important element to include in the preparation of a Cochrane overview of reviews [19]. Our definition of ‘high-quality’ was a review that addressed at least 7 of the 11 AMSTAR criteria. We deemed that setting a cut-off for the total score to indicate quality was appropriate, as psychometric testing of the AMSTAR tool revealed that, as each component score measures a different domain of quality, the summary score is meaningful [20]. Detailed results of appraisal of all relevant systematic reviews are presented in Additional File 2.

### Data synthesis

Data extracted from the systematic reviews were categorised and presented in tables and forest plots. Summary findings are presented in a narrative synthesis.

## Results

**Fig. 1:**
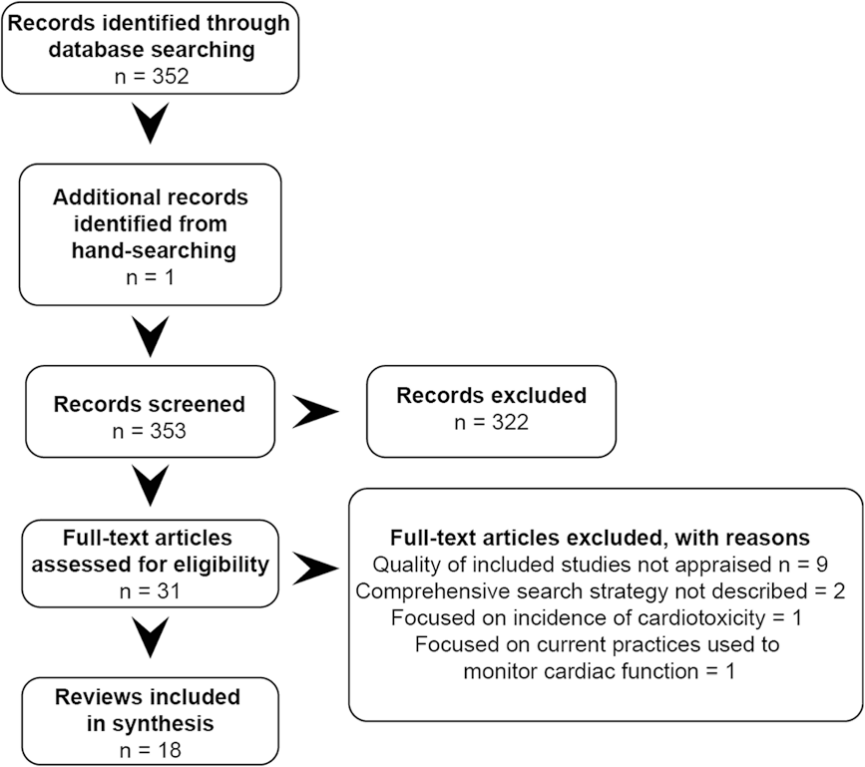
Prisma flow chart - search results

Overall, 31 publications from 352 citations were identified as potentially relevant. Of note, 11 relevant systematic reviews were judged to be of poor quality according to the AMSTAR criteria and were therefore excluded from this meta-review. Eighteen systematic reviews fulfilled the inclusion and exclusion criteria (Fig. 1).

### Systematic review characteristics

The majority of reviews included randomized controlled trials [21–35], with only two reviews (11 %) also including prospective cohort designs [36, 37] (Table 2). The mean number of studies included in the reviews was 14.9 (range = 2–55). The majority of the systematic reviews (*n*-12; 67 %) pooled results from individual studies for meta-analysis [21–26, 28, 30–33]. The reviews that did not use meta-analysis used a narrative approach to synthesise the findings (*n* = 6; 33 %*)* [27, 34–38]. The systematic reviews were published from 2004 to 2013.

**Table 2:** Characteristics of included reviews

Author (Year)	PICO	Characteristics of included studies	Intervention details	Summary of findings	Meta-analysis	AMSTAR score
			**Detection**			
Bryant et al. (2007) [36]	**P:** Children receiving anthracyclines	• One controlled trial and 6 cohort studies	• cTnT	• C-TnT can be used to assess cardioprotection using dexrazoxane	n	7
	**I:** Cardiac markers	• Published from 1983 to 2005	• echocardiography	• ANP and BNP are elevated in children who received anthracyclines		
	**C:** Healthy control group	• Length of follow-up in the studies was not reported	• ANP, BNP	• NT-pro-BNP levels higher in children receiving anthracyclines and had cardiac dysfunction compared to those without		
	**O:** Cardiac damagePublish		• Serum lipid peroxide			
			• Serum carnitine			
			• NT-pro-BNP			
**Prevention of anthracycline-induced cardiotoxicity**						
Van Dalen et al. (2010) [30]	**P:** Cancer patients	• 8 controlled trials	• Doxorubicin vs epirubicin	• No difference in rate of clinical heart failure between epirubicin and doxorubicin (RR = 0.36; 95 % CI = 0.12–1.11)	y	11
	**I:** Anthracycline derivative	• Published from 1984 to 2004	• Doxorubicin vs liposomal-encapsulated doxorubicin	• Lower rate of clinical heart failure (RR = 0.20, 95 % CI 0.05 to 0.75) and subclinical heart failure (RR = 0.38, 95 % CI 0.24 to 0.59) associated with liposomal-encapsulated doxorubicin compared with doxorubicin.		
	**C:** Another anthracycline with the same infusion duration and peak dose. Other chemotherapy and radiotherapy involving the heart region must have been the same as the intervention group.	• Median length of follow-up ranged from 21 to 41 months	• Epirubicin vs liposomal-encapsulated doxorubicin	• No significant difference in the occurrence of clinical and subclinical heart failure between epirubicin and liposomal-encapsulated doxorubicin (RR = 1.13, 95 % CI 0.46 to 2.77, *p* = 0.80).		
	**O:** Anthracycline-induced heart failure, subclinical cardiac dysfunction, abnormalities in cardiac function, tumor response, patient survival, other toxicities, quality of life.					
Van Dalen et al. (2009) [31]	**P:** Cancer patients who received anthracycline chemotherapy	• 11 controlled trials	• Infusion duration	• In meta-analysis of 5 studies with 557 patients, a lower rate of clinical heart failure was observed with an infusion duration of 6 h or longer as compared to a shorter infusion duration (RR = 0.27; 95 % CI = 0.09 to 0.81)	y	11
	**I:** Dosage schedule (different peak dose or infusion duration)	• Published from 1989–2008	• Peak doses (maximal dose received in one week)	• No significant difference in the occurrence of heart failure for different peak doses of anthracyline chemotherapy		
	**C:** Same anthracycline derivative with the same dose. Other chemotherapy and radiotherapy involving the heart region must have been the same as the intervention group.	• Length of follow-up ranged from 7 days to median of 9 years.				
	**O:** heart failure, subclinical cardiac dysfunction, abnormalities in cardiac function, tumor response, patient survival, other toxicities, quality of life.					
Van Dalen et al. (2011) [29]	**P:** Cancer patients	• 18 controlled trials	• N-acetylcysteine	Only dexrazoxane showed a statistically significant cardioprotective effect (Heart failure RR = 0.29; 95 % CI = 0.20–0.41)	y	11
	**I:** Anthracycline with a cardioprotective agent	• 1983–2009	• Phenethylamines			
	**C:** Anthracycline with or without a placebo	• Length of follow-up was not available for most of the included studies	• Coenzyme Q10			
	**O:** Anthracycline-induced heart failure, subclinical cardiac dysfunction, abnormalities in cardiac function, tumor response, patient survival, other toxicities, quality of life.	• In those that reported length of follow-up, it ranged from 6 months up to 5.2 years.	• Combination of vitamin E, vitamin C and Nacetylcysteine			
			• Dexrazoxane			
			• Amifostine			
			• Carvedilol			
			• L-carnitine			
Itchaki et al. 2013 [33]	**P:** advanced follicular lymphoma	• 8 RCT conducted between 1974 and 2011.	• ACR regardless of additional agents, with or without radiotherapy.	• No advantage to ACR in overall survival (HR = 0.99; 95 % CI = 0.77–1.29)	y	11
	**I:** anthacyclines (ACR)	• Length of follow-up ranged from 3 to 5 years in most trials.	• Non-ACR, as a single agent or multiple agents, regardless of dose.	• ACR not significantly better than non-ACR in complete response (RR 1.05;95 % CI 0.94–1.18)		
	**C:** non ACR regardless of dose			• ACR superior to non-ACR in disease control (HR = 0.65; 95 %CI = 0.52–0.81)		
	**O:** overall survival, Progression free survival, Complete response, overall response rate, remission duration, relapse, disease control, Quality of life, adverse events.			Increased risk for cardiotoxicity associated with ACR (RR = 4.55; 95 % CI = 0.92–22.49)		
Smith et al. (2010) [32]	**P:** child and adult patients with Breast or ovarian cancer, sarcoma, non-Hodgkin's or Hodgkin's lymphoma, myeloma	• 55 RCT		**Clinical cardiotoxicity (congestive heart failure)**	y	9
	**I:** anthracycline agent in liposomal or non-liposomal formulation or another non-anthracycline containing chemotherapy regimen	• Studies published between 1985 and 2007	Anthracyclines: doxorubicin, epirubicin, duanorubicin, idarubicin.	• Authors reported that outcomes occurred early and while participants were receiving treatment except in one study where it was not clear when cardiotoxicity occurred.		
	**C:** anthracycline agent	• Length of follow-up not summarised		• Anthracycline vs no anthracycline (OR 5.43; 95 % CI = 2.34–12.62)		
	**O:** Clinical cardiotoxicity (diagnosis of chronic heart failure)			• Bolus versus continuous infusion (OR = 4.13; 95 % CI = 1.75–9.72)		
	Subclinical cardiotoxicity (Reduction in left ventricular ejection fraction or abnormality in cardiac function determined using a diagnostic test)			• Liposomal doxorubicin vs doxorubicin (OR = 0.18; 95 % CI = 0.08–0.38)		
				• Epirubicin vs doxorubicin OR = 0.39 (95 % CI = 0.2–0.78)		
				• Anthracycline vs mitoxantrone OR = 2.88 (95 % CI = 1.29–6.44)		
				• Dexrazoxane vs no dexrazoxane OR = 0.21 (95 % CI = 0.13–0.33)		
				• Anthracycline was associated with increased risk of sub-clinical cardiotoxicity (OR = 6.25; 95 % CI = 2.58–15.13).		
				• Rate of cardiac deaths in 4 studies was significantly higher in the anthracycline groups (OR = 4.94; 95 % CI = 1.23–19.87, *p* = 0.025).		
**Dietary supplementation**						
Roffe et al. (2004) [34]	**P:** Cancer patients	• 6 controlled trials	Dose ranged from 30 mg per day to 240 mg per day	• Significant differences between groups observed in various ECG measures.	n	7
	**I:** Coenzyme Q10	(1 placebo-controlled, double-blinded study, 5 open label)		• Effect on heart failure or subclinical cardiac dysfunction was not reported in the trials		
	**C:** Any comparison	• Published between 1982 and 1996				
	**O:** All outcomes considered	• Length of follow-up was not reported				
**Prevention of cardiotoxicity associated with prostate cancer treatment**						
Shelley et al. (2008) [27]	**P:** Hormone-refractory prostate cancer	• 47 RCT published between 1977 and 2005	Drug categories included:	• Severe cardiovascular toxicity was more common with Estramustine versus Best Supportive Care or Hormones.	n	10
	**I:** Chemotherapy	• Length of follow up was not reported	• estramustine,	• Similar rates of cardiotoxicity with estramustine alone and medroxyprogesterone acetate plus epirubicin.		
	**C:** Any comparison		• 5-fluorouracil	• Cardiotoxicity was less common with epirubicin (11 %) than doxorubicin (48 %).		
	**O:** Overall survival, Disease-specific survival, PSA response, time to progression, pain response, toxicity, quality of life.		• cyclophosphamide	• Doxorubicin combined with diethlystilbestrol was more cardiotoxic than doxorubicin (7 % vs 1 %).		
			• doxorubicin			
			• mitoxantrone			
			• docetaxel			
**Prevention in children**						
Bryant et al. (2007) [35]	**P:** Children receiving anthracyclines	• 4 controlled trials published between 1994 and 2004	• Infusion versus rapid bolus infusion	• No cost-effectiveness data were identified in the systematic review	n	7
	**I:** Any cardioprotection intervention	• Length of follow-up ranged from 25 to 56 months	• Coenzyme Q10	• There were conflicting results in trials of rapid or continuous infusion of anthracycline chemotherapy		
	**C:** Any comparison		• Dexrazoxane	• Coenzyme Q10 was examined in one small trial (n = 20).		
	**O:** Mortality, heart failure, arrhythmia, measures of cardiac function and cost-effectiveness			• Mean reduction in percentage left ventricular fraction shortening was lower in the group that received coenzyme Q10.		
				• Dexrazoxane was examined in a trial with 105 participants.		
				• Fewer patients who received dexrazoxane had elevations in troponin (21 % vs 50 %; *p* < 0.001)		
Sieswerda et al. 2011 [37]	**P:** children with cancer	• 15 observational studies published between 1998 and 2007	• Different liposomal anthracyclines looked at Liposomal daunorubicin, pegylated liposomal doxorubicin, liposomal doxorubicin.	No evidence from controlled trials was identified.	n	7
	**I:** liposomal anthracyclines	• (9 prospective cohort studies, 2 retrospective cohort studies, three case reports, one unclear design)		Impossible to know whether there are differences in outcomes		
	**C:** Any comparison	• Duration of follow up was reported in 10 studies (ranged from 1 to 58 months)				
	**O:** cardiotoxicity, tumour response, adverse events					
Van dalen et al. 2012 [28]	**P:** children with cancer	• 8 RCT published from 1975 to 2009	1153 treatment, 1121 control.	• Rate of cardiac death was similar between treatment groups in meta-analysis of two trials (RR = 0.41; 95 % CI = 0.04–3.89)	y	11
	**I:** anthracyclines	• Length of follow-up was not mentioned in the majority of trials	Culmulative duanorubicin treatment protocol 90–350 mg/m2.	• No significant difference in HF between treatment groups in one trial (RR = 0.33; 95 % CI = 0.01–8.02)		
	**C:** non anthracycline		Peak dose of anthracycline in one week = 25–90 mg/m2. doxorubicin treatment protocol was 300–420 mg/m2.			
	**O:** survival		Peak dose doxorubicin in 1 week 25–60 mg/m2			
	Tumour response cardiotoxicity					
**Prevention of cardiotoxicity associated with breast cancer treatment**						
Valachis et al. (2013) [24]	**P:** Breast cancer	• 6 controlled trials that were all published in 2012.	Anti-HER2 monotherapy (lapatinib or trastuzumab or pertuzumab)	• Pooled OR for CHF in patients with breast cancer receiving dual anti-HER2 therapy versus anti-HER2 monotherapy was 0.58 (95 % CI: 0.26–1.27, *p*-value = 0.17)	y	8
	**I:** anti-HER2 monotherapy	• Length of follow-up was not reported.		• Pooled OR of LVEF decline with dual anti-HER2 therapy versus anti-HER2 monotherapy was 0.88 (95 % CI: 0.53–1.48, *p*-value = 0.64)		
	**C:** anti-HER2 combination therapy			• Comparable cardiac toxicity between these two therapies		
	**O:** LVEF decline less than 50 % or more than 10 % from baseline, National Cancer Institute Common Toxicity Criteria Chronic heart failure grade 3 or more.					
Viani et al. 2007	**P:** HER-2-positive early breast cancer	• 5 RCT published in 2005 and 2006	Doxorubicin and cyclophosphamide (AC) + paclitaxel (P).	• Meta-analysis of 5 trials of adjuvant trastuzumab revealed a significant reduction in mortality (p < 0.00001), recurrence (p < 0.00001), metastases (p < 0.00001) and second tumours (p =0.007) compared with no trastuzumab	y	10
	**I:** adjuvant trastuzumab	• Length of follow-up ranged from 9 to 60 months after randomisation	Docetaxel or vinorelbine + fluorouracil, epirubicin and cyclophosphanide.	• Increased cardiotoxicity including symptomatic cardiac dysfunction and asymptomatic decrease in LVEF with trastuzumab compared to no trastuzumab		
	**C:** any comparison		Doxo, cyclo + trastuz.	• The likelihood of cardiac toxicity was 2.45 times higher for trastuzumab compared with no trastuzumab (statistically significant heterogeneity)		
	**O:** mortality, recurrance, metastases, second tumour no breast cancer rate		Docetaxel, carboplatin + trastuz.			
	Cardiac toxicity and brain metastases		AC + docetaxel.			
Qin et al. 2011 [21]	**P:** node negative breast cancer	• 19 RCT published from 2003 to 2010	Taxane treatment vs non taxane treatment	• Disease free survival: taxane treatment HR 0.82, 95 % CI 0.76–0.88	y	10
	**I:** adjuvant taxane	• Median length of follow-up ranged from 35 to 102 months		• Overall Survival: HR 0.85, 95 % CI 0.78–0.92 favoured taxane		
	**C:** chemo without taxane			• increased toxicity for neutropenia (OR = 2.28, 95 % CI 1.25–4.16), fatigue (OR = 2.10, 95 % CI 1.37–3.22), diarrhea (OR = 2.16, 95 % CI 1.32–3.53), stomatitis (OR 1.68, 95 % CI 1.04–2.71), oedema (OR 6.61, 95 % CI 2.14–20.49).		
	**O:** disease free survival, overall survival, drug related toxicityof taxane			• In pooled analysis of results from 7 trials, there was no statistically significant difference in the rate of cardiotoxicty between chemotherapy regimens with or without taxanes (OR 0.95; 95 % CI = 0.67–1.36)		
				• taxane treatment showed significant reduction in death and recurrence		
Lord et al. 2008 [26]	**P:** metastatic breast cancer	• 34 RCT published between 1974 and 2004	• Comparison between anthracyclines and non-antitumour antibiotic regimens.	• 23 trials with 4777 patients that compared anthracycline with non-antitumour antibiotic regimens reported on cardiotoxicity.	y	10
	**I:** anti-tumour antibiotics	• Length of follow-up was not reported in most trials	• Comparison between mitoxantrone and non-anti-tumour antibiotic regimen	• Patients who received anthracyclines were more likely to develop cardiotoxicity OR = 5.17 (95 % CI = 3.16–8.48)		
	**C:** chemo regimens without anti tumour antibiotics	• Estimated length of follow-up from survival curves ranged from 2 to 102 months.		• Overall survival was reported in 23 studies of anthracyclines. No statistically significant difference in overall survival was observed between the regimens (HR 0.97, 95 % CI 0.91–1.04)		
	**O:** overall survival, time to progression, response, quality of life, toxicity			• The rate of cardiotoxicty was not reported in the mitoxantrone comparison.		
Ferguson et al. 2007 [22]	**P:** breast cancer	• 12 RCT published from 2002 to 2006	Any taxane contain regime vs regimen without taxane	• No difference in the risk of developing cardiotoxicity between taxane containing and non-taxane containing regimens (OR 0.90, 95 %CI 0.53 to 1.55) in meta-analysis of 6 studies involving 11557 patients.	y	11
	**I:** chemotherapy with taxane	• Length of follow-up was 43 to 69 months.				
	**C:** chemotherapy without taxane					
	**O:** overall survival**,** disease free survival, toxicity, quality of life, cost effectiveness					
Duarte et al. 2012 [25]	**P:** breast cancer	• 4 RCT published between 2003 and 2009	Combinations Taxane and anthracycline; anthracycline; combined neo-adjuvant and adjuvant chemo; adjuvant vs non-adjuvant therapy; granulocyte colony-stimulation factor; adjuvant tamoxifan prescribed for 5 years	• Disease free survival: dose dense therapy significant improvement (HR = 0.83; 95 % CI = 0.73–0.95)	y	9
	**I:** conventional chemotherapy	• Length of follow-up ranged from 23 to 125 months		• Dose dense chemotherapy not capable of improving overall survival (HR = 0.86; 95 % CI 0.73–1.01).		
	**C:** aggressive adjuvant chemo			• Women who received a dose-dense chemotherapy regimen were not more likely to develop cardiotoxicity (OR = 0.5; 95 % CI = 0.05–5.54).		
	**O:** overall survival, disease free survival, incidence of Common Toxicity Criteria Scale grades 3,4,5					
**Management**						
Sieswerda et al. 2011 [38]	**P:** children with cancer	2 RCT published in 2004 and 2008	• Enalapril Vs placebo	• 203 patients in total	n	11
	**I:** anthracycline induced cardiotoxicity medical interventions		• Phosphecreatine vs control treatment (vitamin C, adenosine tri-phosphate, vitamin E, oral co-enzyme Q10)	**Enalapril trial**		
	**C:** placebo, other medical interventions, no treatment			• Median follow-up was 2.8 years		
	**O:** overall survival, mortality due to HF, development of HF, adverse events and tolerability			• One intervention participant developed clinically significant decline in cardiac performance compared with 6 control participants (RR = 0.16, 95 % CI 0.02–1.29).		
				• Higher occurrence of dizziness or hypotension (RR 7.17, 95 % CI 1.71 to 30.17) associated with enalapril		
				• Higher occurrence of fatigue associated with enalapril (p = 0.013).		
				**Phosphocreatine trial**		
				• Length of follow-up estimated to be 15 days		
				• No deaths in both groups		
				• No adverse events reported		
				• no definitive conclusions can be drawn due to small sample size		

Legend: *cTnT* Cardiac Troponin T, *ANP* Atrial Natriuretic Peptide, *NT-BNP* N-terminal Brain Natriuretic Peptide, *ACR*, anthacyclines, *LVEF* Left ventricular ejection fraction, *HF* Heart failure, *95 % CI* 95 % Confidence Interval, *RR* Relative risk, *OR*, Odds ratio, *HR* Hazard ratio, *RCT* Randomised controlled trial.

### Key findings from systematic reviews

#### Detection of cancer treatment-induced cardiotoxicity

Only one systematic review focused on interventions to detect cancer treatment-induced cardiotoxicity [36]. This systematic review identified one randomized controlled trial and six cohort studies that investigated the role of cardiac biomarkers, such as brain natriuretic peptide, in the early detection of cardiotoxicity in children who received anthracycline therapy [36]. The authors reported that the overall quality of the evidence was poor, due to a lack of randomized controlled trials and small sample sizes [36]. Based on these findings, the authors of the systematic review concluded that no clear recommendations for practice could be made regarding the use of cardiac biomarkers for the early detection of anthracycline-induced cardiotoxicity [36]. However, it is important to note that this review was published in 2007, with the literature search only current to January 2006.

#### Prevention of cancer treatment-induced cardiotoxicity

The majority (n = 16; 89 %) of the systematic reviews investigated strategies to prevent cancer treatment-induced cardiotoxicity [21–35, 37]. These reviews were further categorized into the following: Prevention of

1. Cardiotoxicity specifically associated with breast cancer treatment [21–26]
2. Cardiotoxicity specifically associated with prostate cancer treatment [27];
3. Anthracycline-induced cardiotoxicity in adult cancer patients [28, 30–33]
4. Cardiotoxicity through dietary supplementation [34]; and
5. Cancer treatment-induced cardiotoxicity in children [28, 35, 37].

Prevention-focused systematic reviews reported clinical cardiotoxicity, defined as the diagnosis of heart failure by a physician or a decline in left ventricular ejection fraction below 40 %, and sub-clinical cardiotoxicity. Definitions of sub-clinical cardiotoxicity varied considerably across reviews. For example, reviews used histological [30, 31], electrocardiographic [34] or echocardiographic [30–32] measurements to identify the presence of myocardial necrosis as a marker of sub-clinical cardiotoxicity.

**Fig. 2:**
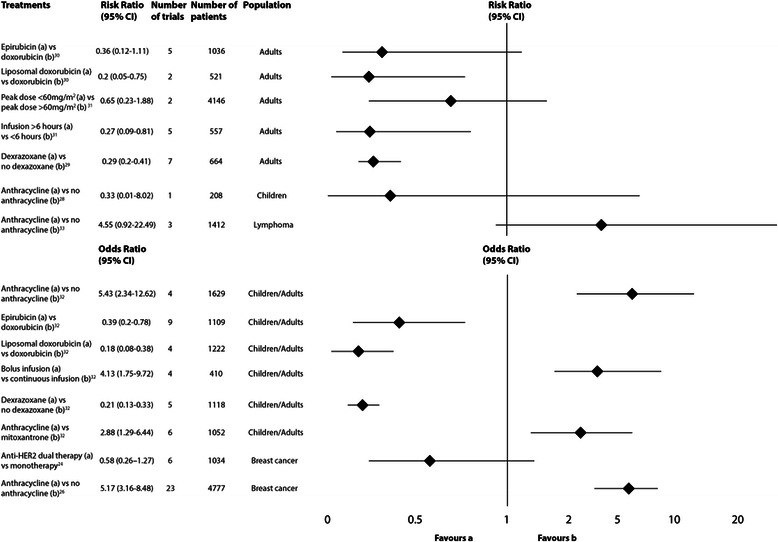
Summary of meta-analyses of included systematic reviews with clinical heart failure as the outcome

The forest plot presented in Fig. 2 displays the results from meta-analyses that examined the effectiveness of different chemotherapy regimens or cardioprotective agents in the prevention of clinical cardiotoxicity. Differences between systematic reviews in their definition of what constituted sub-clinical cardiotoxicity precluded the formation of a similar figure for this outcome.

#### Prevention of cardiotoxicity associated with breast cancer treatment

Two of the breast cancer systematic reviews focused on taxane-based chemotherapy [21, 22]. In one pooled analysis of the results of 7 trials, there was no statistically significant difference in the rate of cardiotoxicity between adjuvant chemotherapy regimens with or without taxanes in women with early or operable breast cancer (OR 0.95; 95 % CI = 0.67–1.36) [21]. An earlier systematic review, which also examined the adverse effects of taxane-based adjuvant chemotherapy in women with early breast cancer, produced similar results [22]. Meta-analysis of 6 trials including 11,577 patients of adjuvant chemotherapy including a taxane revealed that the risk for development of cardiotoxicity was 11 per 1,000 (95 % CI = 6–18) [22]. In comparison, the risk for cardiotoxicity in women with early breast cancer who received adjuvant chemotherapy without a taxane was 12 per 1,000 [22]. The relative risk was 0.9 (95 % CI = 0.53–1.54) [22]. Of note, the chemotherapy regimens of control and intervention arms of the studies included in the meta-analyses contained anthracyclines [21, 22]. As there were no differences in the rate of cardiotoxicity between participants who did and did not receive taxanes, it would appear that the rates of cardiotoxicity were likely due to the use of anthracyclines.

A specific focus of two further systematic reviews that examined the prevention of cardiotoxicity associated with breast cancer treatment investigated the impact of anti-human epidermal growth factor receptor 2 (HER-2) therapy [23, 24]. One systematic review aimed to determine whether there was an increased risk of cardiotoxicity in breast cancer patients treated with dual HER-2 blockade (pertuzumab plus trastuzumab, or trastuzumab plus lapatanib) compared to monotherapy (lapatanib or trastuzumab or pertuzumab) [24]. No statistically significant difference in the likelihood of developing clinical heart failure or of decline in left ventricular ejection fraction was identified [24]. The authors concluded that the evidence supported the use of dual therapy in this population with no adverse cardiac effects. The second breast cancer systematic review focused on the anti-HER-2 medication trastuzumab [23]. Meta-analysis indicated that the odds of developing cardiotoxicity were 2.45 times higher (95 % CI = 1.89–3.16) in subjects prescribed trastuzumab [23]. However, overall mortality, recurrence and mortality rates were decreased in subjects who received trastuzamab despite their higher odds of developing post-treatment cardiac symptoms. [23] Close monitoring of cardiac function was recommended, based on these findings.

A systematic review of randomized controlled trials of dose-dense anthracycline-based chemotherapy in early breast cancer, comprising a meta-analysis that combined n = 1,310 patients, revealed that women who received a dose-dense chemotherapy regimen were not more likely to develop cardiotoxicity (OR = 0.5; 95 % CI = 0.05–5.54) [25]. Trials of dose-dense chemotherapy were defined in this review as the same type and total amount of the drug administered over a shorter interval of time [25]. A further systematic review focused on anthracycline regimens for metastatic breast cancer [26]. The authors recommended careful consideration regarding the use of anthracyclines in this population due to the increased likelihood of developing cardiotoxicity (OR = 5.17; 95 % CI = 3.16–8.48) [26].

#### Prevention of cardiotoxicity associated with prostate cancer treatment

One systematic review addressed the issue of cardiotoxicity associated with treatment for hormone-refractory prostate cancer [27]. While 47 trials met the inclusion criteria for this review, the chemotherapy regimens investigated were too dissimilar to conduct meta-analysis [27]. No specific recommendations for the prevention of cardiotoxicity in this population were noted by the authors of this review.

#### Prevention of anthracycline-induced cardiotoxicity in adult cancer patients

In a systematic review of cardioprotective agents used during anthracycline therapy, the authors concluded that of the eight different agents for which there were randomized controlled trials, only dexrazoxane could be recommended for clinical practice [29]. Conflicting recommendations were provided in another systematic review by Smith *et al.* [32], which concluded that the evidence was not sufficiently robust to support the routine use of any particular cardioprotective agent, nor a liposomal formulation or alternative anthracycline treatment regimen. In regard to the use of anthracycline derivatives, a 2010 systematic review by van Dalen *et al.* came to a similar conclusion, suggesting that further research is needed to provide recommendations regarding the use of these alternative chemotherapy regimens [30].

Anthracycline administration considerations were examined for their potential association with cardiotoxicity in a second systematic review by van Dalen [31]. This review identified that continuous anthracycline infusion (>6 h) rather than bolus injection reduced the risk of cardiotoxicity [31]. No differences were observed in the rate of cardiotoxicity as a result of different doxorubicin peak doses [31].

A recently published review (2013) by Itchaki focused on anthracycline use in people receiving treatment for advanced follicular lymphoma [33]. Due to the increased risk ratio for cardiotoxicity (4.55; 95 % CI = 0.92-22.49) associated with anthracycline treatment in this population, the authors concluded that evidence of the benefit of anthracyclines in this population is limited [33].

#### Dietary supplementation

One systematic review appraised randomized and non-randomized studies that reported the use of coenzyme Q10 (CoQ10) to reduce the adverse effects of cancer treatment [34]. Only three randomized controlled trials, which included a total of 140 patients, investigated the effects of CoQ10 on cardiotoxicity [34]. These trials were not subjected to meta-analysis. The authors of the systematic review concluded that CoQ10 could provide some protection against cardiotoxicity during cancer treatment based on the fact that significant differences in electrocardiographic measurements were identified between control and CoQ10 groups [34]. However, using CoQ10 in clinical practice was not recommended, due to insufficient data [34].

#### Prevention of cancer treatment-induced cardiotoxicity in children

All of the systematic reviews that focused on the prevention of cardiotoxicity in children addressed this issue as it related to anthracycline-based chemotherapy. Sieswerda *et al.* [37] concluded that randomized controlled trials are needed to increase understanding of the benefits and risks of liposomal anthracyclines in children, as the evidence to date solely consists of observational studies [37]. In a further systematic review, meta-analysis of two randomized controlled trials revealed no statistically significant difference in the risk of cardiac death (RR = 0.4; 95 %CI = 0.04–3.89) or heart failure (RR = 0.33; 95 %CI = 0.01–8.02) in children who received anthracyclines [28]. However, the total number of participants in the randomized controlled trials was small (n = 410) [28]. As such, no firm conclusions regarding the implications for clinical practice were drawn from this analysis. A further systematic review focused on cardioprotection in children who received anthracyclines [35]. Based on the fact that only four randomized controlled trials with methodological limitations met the inclusion criteria, the authors concluded that there was limited evidence to guide cardioprotective therapies in this population and definitive recommendations for practice could not be made [35].

#### Management of cancer treatment-induced cardiotoxicity

Only one systematic review focused on interventions to treat cancer treatment-induced cardiotoxicity [38]. This review focused on the treatment of anthracycline-induced cardiotoxicity in children. Only two randomized controlled trials, which enrolled a total of 203 patients, were included in this review. The two interventions tested were enalapril and phosphocreatine. While the participants who received enalapril were less likely to experience decline in cardiac function, the difference between groups was not statistically significant (*p* < 0.5). Moreover, participants who received enalapril were more likely to experience hypotension, dizziness and fatigue. Therefore, the authors concluded that the benefits of this therapy be weighed against the greater risk of side effects in children with asymptomatic cardiotoxicity [38]. Conclusions regarding the use of phosphocreatine could not be made due to the high risk of bias. The authors of the review concluded that further high quality randomized controlled trials are required in this field [38].

## Discussion

This aim of this meta-review was to appraise and synthesise the systematic reviews that have focused on the prevention, early detection and management of cancer treatment-induced cardiotoxicity in order to aid policy and practice decision-making. Based on the 18 systematic reviews included in this meta-review that were deemed to be high quality according to the AMSTAR criteria, the following conclusions can be drawn. First, there is insufficient evidence to draw firm conclusions regarding the utility of cardiac biomarkers for the detection of cancer treatment-induced cardiotoxicity. Based on conclusions drawn from systematic reviews focused on prevention, the following strategies could reduce the risk of cardiotoxicity: 1) The concomitant administration of dexrazoxane with anthracylines; 2) The administration of continuous anthracyclines, preferably for longer than 6 h, rather than bolus dosing; and 3) The administration of anthracycline derivatives such as epirubicin or liposomal-encapsulated doxorubicin instead of doxorubicin. In this context, it should be noted that while dexrazoxane is listed in the relevant pharmaceutical benefits scheme in some countries for this indication (*e.g.* it is listed as such by the FDA in the USA), in others such as Australia it is not. Hence its routine use would be problematic in some countries, as it would be hard to procure and expensive for patients who already incur considerable treatment overheads. In addition, in many high volume chemotherapy facilities it is not logistically possible to deliver anthracyclines over an extended period. In the facilities in which this review team work, for example, 30 min of infusion via a 100 ml minibag is the norm for reasons of economy and patient throughput.

There is limited evidence pertaining to the effectiveness of interventions to manage cancer treatment-induced cardiotoxicity. While two different medical interventions were identified in a systematic review that focused on treatment strategies for cardiotoxicity in childhood cancer (enalapril and phosphocreatine), neither was associated with statistically significant improvement in ejection fraction or mortality.

The largest number of systematic reviews included in this meta-review addressed the prevention of cancer therapy-induced cardiotoxicity. As demonstrated in Fig. 2, few strategies appear to reduce the risk of developing clinical heart failure. These included the avoidance of anthracycline-based chemotherapy (which is routine where cardiac risk before therapy is known), the use of doxorubicin derivatives, a longer anthracycline infusion duration and concomitant administration of the cardioprotective agent dexrazoxane. Of note, all meta-analyses that revealed statistically significant reductions in the rate of clinical heart failure related specifically to the use of anthracyclines. This is not surprising, considering anthracyclines are the focus of the greatest amount of research in this particular field [7]. However, our meta-review identified that the Level 1 evidence from meta-analyses focused on the prevention of cardiotoxicity was derived from a relatively small number of trials and in most cases, less than one thousand participants in total. Therefore, despite the fact that the cardiotoxic effects of this particular chemotherapy regimen have been known for a considerable time, there are still gaps in the evidence regarding how to facilitate early detection and management. In particular, the evidence for strategies that protect children with cancer from developing cardiac complications associated with their treatment is lacking [35].

Of note, one previous overview of systematic reviews on the topic of cancer treatment-induced cardiotoxicity has been published [39]. However, this review was smaller in scope than the present review. It focused only on the prevention of cardiotoxicity associated with anthracycline treatment in the paediatric population [39]. Furthermore, only reviews registered by the Cochrane Collaboration were included in van Dalen et al’s systematic review [39]. Excluding all other reviews is an effective strategy to ensure only high quality systematic reviews when detailed quality appraisal is not employed as part of the meta-review process [40]. It is possible however that systematic reviews not registered with the Cochrane Collaboration will meet many AMSTAR criteria, indicating that sufficient processes were undertaken to ensure potential sources of bias associated with the systematic review process were avoided. Therefore, including only Cochrane reviews in a meta-review is not the optimal choice when quality appraisal is included as part of the meta-review process.

In regard to the quality of the systematic reviews that reported data on cardiotoxicity, this meta-review identified that: 1) the methodology used in a considerable number of systematic reviews was poor (n = 11; 35 % of the potentially relevant reviews were excluded due to low quality according to the AMSTAR criteria); and 2) half of the systematic reviews not registered with the Cochrane Collaboration were of high quality (n = 9; 50 % of reviews that met more than 7 of the AMSTAR criteria were not Cochrane reviews). Based on these findings, it is recommended that future meta-reviews that focus on the prevention, detection and management of cancer treatment-induced toxicities should not include only Cochrane reviews, as high-quality systematic reviews that potentially contain unbiased and important recommendations for practice could be overlooked. However, quality appraisal of the systematic reviews would be required to ensure biased conclusions from systematic reviews that have used poor methodology are avoided.

Specific deficiencies in Level 1 evidence for the detection, prevention and management of cancer therapy-induced cardiotoxicity were identified in this meta-review. Only one high quality systematic review of dietary supplementation was identified, which was published in 2004. Recommendations for practice regarding interventions for the detection of cancer treatment-induced cardiotoxicity were not able to be drawn from this meta-review. However, we have identified that an updated systematic review focusing on the detection of cardiotoxicity is required to help inform clinical practice, as the only previous high quality review included evidence up to January 2006. No Level 1 evidence is available to guide clinical decision-making regarding the prevention, detection or management of radiation-induced cardiotoxicity. While the role of chest irradiation in inducing cardiotoxicity has been known for some time, studies to date have focused on minimizing the dose of radiation to the heart that are not powered to detect clinical differences in the rate of cardiotoxicity [14]. The small number of primary research studies undertaken to investigate strategies to prevent radiation-induced cardiotoxicity is likely the reason why no systematic reviews were identified in our literature search. While an evidence base about the potential effectiveness of exercise as an intervention to aid prevention of cancer treatment-induced cardiotoxicity is also emerging, similarly, no systematic reviews of the effectiveness of this intervention were identified in our comprehensive search of the literature. Based on the positive results observed in animal studies, it is likely that human clinical trials of exercise for the prevention of cardiotoxicity associated with cancer treatment will be reported in the future [41].

### Limitations

It should be noted that only English language reviews were included in our meta-review. However, we considered this to be acceptable because sensitivity testing regarding information published in languages other than English has shown that English language reviews represent a robust view of the available evidence base in health areas [35]. A considerable strength of this meta-review is that we were able to reduce the risk of bias from our conclusions regarding the prevention, detection and management of cancer-treatment induced cardiotoxicity by including only systematic reviews that had considered the quality of included studies in making decisions about the validity of the evidence, as well as the suitability of the included trials for meta-analyses. No attempts were made to combine data from multiple systematic reviews, due to the substantial degree of heterogeneity between the populations, interventions and outcomes investigated. As is the case for all meta-reviews, it should be noted that evidence from recent studies that were not included in the systematic reviews was not able to be considered in our review. For this reason, the majority of evidence regarding the detection, prevention and management of cancer treatment-induced cardiotoxicity included in this meta-review is from studies conducted at least 5 years ago. Another important point to note is that absolute risk of cardiotoxicity was not reported in meta-analyses due to heterogeneity between individual studies.

## Conclusion

This meta-review has highlighted the paucity of high level evidence to guide clinical practice decision-making regarding the detection and management of cancer treatment associated cardiotoxicity. There is a greater amount of evidence available to guide practice in regard to the prevention of this adverse effect of cancer treatment. It is important to note, however, that the meta-analyses that revealed statistically significant reductions in clinical cardiotoxicity only applied to anthracycline based chemotherapeutic regimens. No high-level evidence is available to guide clinical decision-making regarding the prevention, detection or management of radiation-induced cardiotoxicity.

## Additional files

Below is the link to the electronic supplementary material.

Additional file 1: **MEDLINE search strategy.**

Additional file 2: **AMSTAR score of potentially relevant systematic reviews.**

## Abbreviations

CoQ10: Coenzyme Q10
HER-2: Anti-human epidermal growth factor receptor 2
cTnT: Cardiac troponin T
ANP: Atrial natriuretic peptide
NT-BNP: N-terminal nrain natriuretic peptide
ACR: Anthacyclines
LVEF: Left ventricular ejection fraction
HF: Heart failure
95 % CI: 95 % Confidence interval
RR: Relative risk
OR: Odds ratio
HR: Hazard ratio
