# Supplementary material for: The prevention, detection and management of cancer treatment-induced cardiotoxicity: a meta-review
Source: BMC Cancer. 2015 May 7;15:366. doi: 10.1186/s12885-015-1407-6 (PMC4427936; doi:10.1186/s12885-015-1407-6)
Supplement: Supplementary file 1 — MEDLINE search strategy. [file 12885_2015_1407_MOESM1_ESM.docx]

**Additional File 1**

**MEDLINE Search Strategy**

| **Search terms** |
| --- |
| 1 exp Radiotherapy/ 2 exp Radiation Oncology/ 3 (radioth$ or radiat$ or irradiat$ or radiochemo$ or chemoradi$).mp. 4 (1 OR 2 OR 3) 5 exp [Antineoplastic Combined Chemotherapy Protocols](javascript:XslPostBack('ctl00$ctl00$MainContentArea$MainContentArea$ctrlResults','meshDetail','index%7C2%24term%7CAntineoplastic%20Combined%20Chemotherapy%20Protocols%24cmd%7CmeshDetail');)/  6 exp [Maintenance Chemotherapy](javascript:XslPostBack('ctl00$ctl00$MainContentArea$MainContentArea$ctrlResults','meshDetail','index%7C6%24term%7CMaintenance%20Chemotherapy%24cmd%7CmeshDetail');)/  7 exp [Induction Chemotherapy](javascript:XslPostBack('ctl00$ctl00$MainContentArea$MainContentArea$ctrlResults','meshDetail','index%7C7%24term%7CInduction%20Chemotherapy%24cmd%7CmeshDetail');)/  8 exp [Consolidation Chemotherapy](javascript:XslPostBack('ctl00$ctl00$MainContentArea$MainContentArea$ctrlResults','meshDetail','index%7C8%24term%7CConsolidation%20Chemotherapy%24cmd%7CmeshDetail');)/  9 exp [Chemotherapy, Adjuvant](javascript:XslPostBack('ctl00$ctl00$MainContentArea$MainContentArea$ctrlResults','meshDetail','index%7C9%24term%7CChemotherapy%2C%20Adjuvant%24cmd%7CmeshDetail');)/  10 exp [Antineoplastic Agents](javascript:XslPostBack('ctl00$ctl00$MainContentArea$MainContentArea$ctrlResults','meshDetail','index%7C10%24term%7CAntineoplastic%20Agents%24cmd%7CmeshDetail');)/  11 (5 OR 6 OR 7 OR 8 OR 9 OR 10)  12 (4 OR 11)  13 (heart OR heart diseases OR heart disease OR disease, heart OR diseases, heart OR cardiac diseases OR cardiac disease OR diseases,  cardiac OR disease, cardiac OR cardiotoxicity OR cardiomyopathy OR heart failure, congestive OR heart failure OR cardiomyopathy,  congestive OR ventricular dysfunction OR ventricular dysfunction, left OR ventricular dysfunction, right).mp 14 (12 and 13)  15 Systematic review*  16 Meta-analys*  17 (15 OR 16)  18 (14 and 17) |
