# Supplementary material for: The prevention, detection and management of cancer treatment-induced cardiotoxicity: a meta-review
Source: BMC Cancer. 2015 May 7;15:366. doi: 10.1186/s12885-015-1407-6 (PMC4427936; doi:10.1186/s12885-015-1407-6)
Supplement: Supplementary file 2 — AMSTAR score of potentially relevant systematic reviews. [file 12885_2015_1407_MOESM2_ESM.docx]

**Additional File 2**

| **AMSTAR score of potentially relevant systematic reviews** | | | | | | | | | | | | | |
| --- | --- | --- | --- | --- | --- | --- | --- | --- | --- | --- | --- | --- | --- |
| **Author (Year)** | **A priori design** | **Independent data extractors** | **Comprehensive literature search** | **Status of publication as inclusion criteria** | **List of included and excluded studies** | **Characteristics of the included studies** | **Documented quality of included studies** | **Appropriate use of study quality in conclusions** | **Appropriate methods** | **Assessed publication bias** | **Conflict of interest stated** | **AMSTAR total** | **Exclude based on quality** |
| Bryant  Et al 2007[^1^](#_ENREF_1) | n | y | y | Y | N | y | y | y | c/a | n | y | 7 | N |
| Byrant et al 2007[^2^](#_ENREF_2) | n | y | y | y | n | y | y | y | c/a | n | y | 7 | N |
| Magnus et al 2008[^3^](#_ENREF_3) | Y | c/a | Y | Y | N | Y | N | N | N | N | Y | 5 | y |
| Cappone et al 2007[^4^](#_ENREF_4) | Y | Y | Y | Y | N | Y | N | N | Y | N | Y | 6 | Y |
| Lemos Duarte et al 2012[^5^](#_ENREF_5) | N | Y | Y | Y | Y | Y | Y | Y | Y | N | Y | 9 | N |
| Ferguson et al 2007[^6^](#_ENREF_6) | Y | Y | Y | Y | Y | Y | Y | Y | Y | Y | Y | 11 | N |
| Greenlee et al 2010[^7^](#_ENREF_7) | N | c/a | N | N | N | Y | N | N | N | N | Y | 2 | y |
| Humber et al 2007[^8^](#_ENREF_8) | N | c/a | c/a | c/a | N | Y | Y | Y | Y | c/a | Y | 5 | y |
| Itchaki et al 2013[^9^](#_ENREF_9) | Y | Y | Y | Y | Y | Y | Y | Y | Y | Y | Y | 11 | N |
| Kalam et al 2013[^10^](#_ENREF_10) | N | Y | Y | N | Y | Y | N | N | Y | Y | Y | 7 | Y |
| Kelly et al 2013[^11^](#_ENREF_11) | N | c/a | N | Y | N | Y | N | N | c/a | N | Y | 3 | y |
| Ladas et al 2004[^12^](#_ENREF_12) | c/a | c/a | Y | c/a | N | Y | n | N | c/a | N | Y | 3 | y |
| Lang et al 2011[^13^](#_ENREF_13) | Y | Y | Y | Y | Y | Y | Y | Y | Y | Y | Y | 11 | N |
| Lord et al 2008[^14^](#_ENREF_14) | Y | Y | Y | Y | Y | Y | Y | Y | Y | N | Y | 10 | N |
| Moss et al 2009[^15^](#_ENREF_15) | N | c/a | Y | Y | N | N | N | N | N | N | Y | 3 | y |
| Petrelli et al 2012[^16^](#_ENREF_16) | N | Y | N | N | N | Y | Y | Y | Y | N | Y | 6 | Y |
| Qin et al 2011[^17^](#_ENREF_17) | N | Y | Y | Y | Y | Y | Y | Y | Y | Y | Y | 10 | N |
| Roffe et al 2004[^18^](#_ENREF_18) | N | Y | Y | Y | Y | Y | Y | Y | Y | N | Y | 9 | N |
| Siddhartha et al 2009[^19^](#_ENREF_19) | N | N | Y | Y | N | Y | N | N | Y | N | Y | 5 | y |
| Sieswerda et al 2011[^20^](#_ENREF_20) | N | Y | Y | Y | N | Y | Y | Y | c/a | N | Y | 7 | N |
| Sieswerda et al 2011[^21^](#_ENREF_21) | Y | Y | Y | Y | Y | Y | Y | Y | Y | Y | Y | 11 | N |
| Shelley et al 2008[^22^](#_ENREF_22) | Y | Y | Y | Y | Y | Y | Y | Y | Y | c/a | Y | 10 | N |
| Smith et al 2010[^23^](#_ENREF_23) | N | Y | Y | Y | Y | Y | Y | Y | Y | N | Y | 9 | N |
| Vaklavas et al 2010[^24^](#_ENREF_24) | N | N | N | N | N | Y | N | N | c/a | N | N | 1 | y |
| Viani et al 2007[^25^](#_ENREF_25) | n | y | y | y | y | y | y | y | y | y | y | 10 | N |
| Valachis et al 2013[^26^](#_ENREF_26) | N | Y | Y | Y | N | Y | Y | Y | Y | Y | Y | 9 | N |
| Van dalen et al 2006[^27^](#_ENREF_27) | N | N | N | c/a | N | Y | N | N | Y | N | Y | 3 | y |
| Van dalen et al 2009[^28^](#_ENREF_28) | Y | Y | Y | Y | Y | Y | Y | Y | Y | c/a | Y | 10 | N |
| Van dalen et al 2012[^29^](#_ENREF_29) | Y | Y | Y | Y | Y | Y | Y | Y | Y | Y | Y | 11 | N |
| Van dalen et al 2010[^30^](#_ENREF_30) | Y | Y | Y | Y | Y | Y | Y | Y | Y | N | Y | 10 | N |
| Van dalen et al 2011[^31^](#_ENREF_31) | Y | y | Y | y | Y | y | Y | y | Y | y | Y | 11 | N |

Legend: Y=Yes; N=No; CA= Can’t Answer

1. Bryant J, Picot J, Baxter L, Levitt G, Sullivan I and Clegg A. Use of cardiac markers to assess the toxic effects of anthracyclines given to children with cancer: a systematic review. *European Journal of Cancer*. 2007; 43: 1959-66.

2. Bryant J, Picot J, Baxter L, Levitt G, Sullivan I and Clegg A. Clinical and cost-effectiveness of cardioprotection against the toxic effects of anthracyclines given to children with cancer: a systematic review. *Br J Cancer*. 2007; 96: 226-30.

3. Magnus B, Tomas A, Anders A and Eva Ö. CNOP (mitoxantrone) chemotherapy is inferior to CHOP (doxorubicin) in the treatment of patients with aggressive non‐Hodgkin lymphoma (meta‐analysis). *European Journal of Haematology*. 2008; 80: 477-82.

4. Cuppone F, Bria E, Verma S, et al. Do adjuvant aromatase inhibitors increase the cardiovascular risk in postmenopausal women with early breast cancer. *Cancer*. 2008; 112: 260-7.

5. Lemos Duarte I, da Silveira Nogueira Lima JP, Passos Lima CS and Deeke Sasse A. Dose-dense chemotherapy versus conventional chemotherapy for early breast cancer: a systematic review with meta-analysis. *The Breast*. 2012; 21: 343-9.

6. Ferguson T, Wilcken N, Vagg R, Ghersi D and Nowak AK. Taxanes for adjuvant treatment of early breast cancer. *Cochrane Database Syst Rev*. 2007; 4.

7. Greenlee H, Hershman DL and Jacobson JS. Use of antioxidant supplements during breast cancer treatment: a comprehensive review. *Breast Cancer Research and Treatment*. 2009; 115: 437-52.

8. Humber C, Tierney J, Symonds R, et al. Chemotherapy for advanced, recurrent or metastatic endometrial cancer: a systematic review of Cochrane collaboration. *Annals of Oncology*. 2007; 18: 409-20.

9. Itchaki G, Gafter‐Gvili A, Lahav M, et al. Anthracycline‐containing regimens for treatment of follicular lymphoma in adults. *Cochrane Database Syst Rev*. 2013: Art. No. CD 008909.

10. Kalam K and Marwick TH. Role of cardioprotective therapy for prevention of cardiotoxicity with chemotherapy: a systematic review and meta-analysis. *European Journal of Cancer*. 2013; 49: 2900-9.

11. Kelly C, Bhuva N, Harrison M, Buckley A and Saunders M. Use of raltitrexed as an alternative to 5-fluorouracil and capecitabine in cancer patients with cardiac history. *European Journal of Cancer*. 2013; 49: 2303-10.

12. Ladas EJ, Jacobson JS, Kennedy DD, Teel K, Fleischauer A and Kelly KM. Antioxidants and cancer therapy: a systematic review. *Journal of Clinical Oncology*. 2004; 22: 517-28.

13. Lang D and George C. Acute adverse reactions of rapid Rituximab infusion among adult patients with Non-Hodgkin Lymphoma and Chronic Lymphocytic Leukemia. *The JBI Database of Systematic Reviews and Implementation Reports*. 2011; 9: 1-37.

14. Lord S, Ghersi D, Gattellari M, Wortley S, Wilcken N and Simes J. Antitumour antibiotic containing regimens for metastatic breast cancer. *Cochrane Database Syst Rev*. 2004; 4.

15. Moss LS, Starbuck MF, Mayer DK, Harwood EB and Glotzer J. Trastuzumab-induced cardiotoxicity. *Oncology nursing forum*. 2009; 36: 676-85.

16. Petrelli F, Borgonovo K, Cabiddu M, Lonati V and Barni S. Mortality, leukemic risk, and cardiovascular toxicity of adjuvant anthracycline and taxane chemotherapy in breast cancer: a meta-analysis. *Breast Cancer Research and Treatment*. 2012; 135: 335-46.

17. Qin Y-Y, Li H, Guo X-J, et al. Adjuvant chemotherapy, with or without taxanes, in early or operable breast cancer: a meta-analysis of 19 randomized trials with 30698 patients. *PloS One*. 2011; 6: e26946.

18. Roffe L, Schmidt K and Ernst E. Efficacy of coenzyme Q10 for improved tolerability of cancer treatments: a systematic review. *Journal of Clinical Oncology*. 2004; 22: 4418-24.

19. Siddhartha G and Vijay P. R-CHOP versus R-CVP in the treatment of follicular lymphoma: a meta-analysis and critical appraisal of current literature. *J Hematol Oncol*. 2009; 2: 14.

20. Sieswerda E, Kremer L, Caron H and van Dalen E. The use of liposomal anthracycline analogues for childhood malignancies: A systematic review. *European Journal of Cancer*. 2011; 47: 2000-8.

21. Sieswerda E, van Dalen EC, Postma A, Cheuk D, Caron HN and Kremer L. Medical interventions for treating anthracycline-induced symptomatic and asymptomatic cardiotoxicity during and after treatment for childhood cancer. *Cochrane Database Systematic Reviews*. 2011; Issue 9: CD008011.

22. Shelley M, Harrison C, Coles B, Staffurth J, Wilt TJ and Mason MD. Chemotherapy for hormone-refractory prostate cancer. *Cochrane Database Syst Rev*. 2006; 4.

23. Smith LA, Cornelius VR, Plummer CJ, et al. Cardiotoxicity of anthracycline agents for the treatment of cancer: systematic review and meta-analysis of randomised controlled trials. *BMC Cancer*. 2010; 10: 337.

24. Vaklavas C, Lenihan D, Kurzrock R and Tsimberidou AM. Anti-vascular endothelial growth factor therapies and cardiovascular toxicity: what are the important clinical markers to target? *The Oncologist*. 2010; 15: 130-41.

25. Viani GA, Afonso SL, Stefano EJ, De Fendi LI and Soares FV. Adjuvant trastuzumab in the treatment of her-2-positive early breast cancer: a meta-analysis of published randomized trials. *BMC Cancer*. 2007; 7: 153.

26. Valachis A, Nearchou A, Polyzos NP and Lind P. Cardiac toxicity in breast cancer patients treated with dual HER2 blockade. *International Journal of Cancer*. 2013; 133: 2245-52.

27. van Dalen EC, van den Brug M, Caron HN and Kremer L. Anthracycline-induced cardiotoxicity: comparison of recommendations for monitoring cardiac function during therapy in paediatric oncology trials. *European Journal of Cancer*. 2006; 42: 3199-205.

28. van Dalen EC, Van der Pal H, Caron HN and Kremer L. Different dosage schedules for reducing cardiotoxicity in cancer patients receiving anthracycline chemotherapy. *Cochrane Database Syst Rev*. 2009; 4.

29. Van Dalen E, Raphaël M, Caron H and Kremer L. Treatment including anthracyclines versus treatment not in-cluding anthracyclines for childhood cancer. *Cochrane Database Syst Rev*. 2011: CD006647.

30. van Dalen EC, Michiels E, Caron HN and Kremer L. Different anthracycline derivates for reducing cardiotoxicity in cancer patients. *Cochrane Database Syst Rev*. 2010; 5.

31. van Dalen EC, Caron HN, Dickinson HO and Kremer LCM. Cardioprotective interventions for cancer patients receiving anthracyclines. *Cochrane Database Syst Rev*. 2011.
